# Population distribution and drivers of habitat use for the Burrunan dolphins, Port Phillip Bay, Australia

**DOI:** 10.1002/ece3.11221

**Published:** 2024-04-04

**Authors:** Jemima Beddoe, Jeff Shimeta, Marcel Klaassen, Kate Robb

**Affiliations:** ^1^ School of Science RMIT University Melbourne Victoria Australia; ^2^ Australian Marine Mammal Conservation Foundation Hampton East Victoria Australia; ^3^ School of Life and Environmental Sciences Deakin University Geelong Victoria Australia

**Keywords:** Burrunan dolphin, critically endangered, distribution, habitat use, marine protected area

## Abstract

Bottlenose dolphin (*Tursiops*) populations, also described as the Burrunan dolphins, consist of a resident population of approximately 150 individuals in Port Phillip Bay (PPB), Victoria. Previous reports indicate distribution across a small southern region of PPB; however, little is known about their full distribution patterns across the entire PPB region. Here, we investigate the spatiotemporal distribution of the Burrunan dolphins across four zones representative of PPB benthic habitats and bathymetry to gain a better understanding of the potential drivers of the population's habitat use. Port Phillip Bay, Victoria, Australia. One hundred and twenty‐nine boat‐based surveys were undertaken between March 2015 and August 2021, encompassing 181 sightings. Generalised linear models (GLMs) were used to investigate annual, seasonal and zonal variation. We found no variation in sighting frequencies between years. Austral summer and winter had a significantly higher sighting frequency than autumn. We found that Burrunan dolphins utilise the entire bay, further extending the species range, and show a significantly higher number of sightings in the southern zone than in any other zones. Overlaying dolphin sightings with known oceanographic characteristics within PPB, we found bathymetry and benthic habitats were potential drivers for the Burrunan dolphins distribution and habitat use within the bay, with the dolphins significantly favouring the 5–10 and 10–15 m contour depths. These results show a more widespread distribution across the bay than previously documented. We recommend expansion of the current marine protected areas in the north and south of the bay. This study has increased our understanding of the vital habitat for the Burrunan dolphin populations. By providing evidence‐based conservation recommendations, we hope to improve and contribute to future research, conservation management plans and effective marine protected areas across PPB for the resident Burrunan dolphin population.

## Graphical abstract

The Burrunan dolphin, *Tursiops australis*, a recently described species endemic to south‐eastern Australia, has a resident population of approximately 150 individuals in Port Phillip Bay (PPB), Victoria. Previous reports indicate distribution across a small southern region of PPB; however, little is known about their full distribution patterns across the entire PPB region. This study has increased our understanding of the vital habitat for the Burrunan dolphin population across the whole PPB region, and by providing evidence‐based conservation recommendations we hope to improve and contribute to future research, conservation management plans and effective marine protected areas across PPB for the resident Burrunan dolphin population.

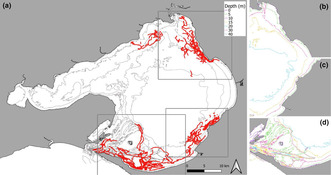

## 1 INTRODUCTION

Marine mammals are a polyphyletic group comprised of approximately 129 species across three orders: Cetacea, Sirenia and Carnivora (Pompa‐Mansilla et al., 2011). Cetaceans (whales, dolphins and porpoises) are amongst the most endangered taxa due to anthropogenic threats. Both coastal and marine habitats are threatened by a combination of anthropogenic impacts, such as the overexploitation of natural resources, habitat loss and degradation, chemical pollution and noise pollution (Marega‐Imamura et al., 2020; Mirimin et al., 2011). Understanding species‐environment relationships is crucial for identifying areas of biological importance and prioritising areas for conservation, marine protected area zoning design, resource management and impact assessment (Elith & Leathwick, 2009; Guisan & Thuiller, 2005; Zanardo et al., 2017). Place‐based protection that is appropriately designated in a critical habitat for particular marine mammal populations can substantially reduce their likelihood of mortality (Hooker et al., 2011). Marine mammals appear to be one of the few groups that have benefitted most from a shift of management practices, away from resource exploitation towards wildlife conservation (Lotze & Milewski, 2004; Lotze & Worm, 2009; Magera et al., 2013).

Assessing spatial distribution, habitat use, site fidelity and the potential drivers for habitat usage allows for the prediction of how individuals might respond to changes in their environment, and provides effective and informed management strategies for endangered marine mammals (Balmer et al., 2013; Prado et al., 2016). Identifying the factors that may influence habitat selection at multiple spatial and temporal scales, such as food availability and predation risk (Heithaus & Dill, 2006), are essential for understanding the drivers of a population's distribution. Marine habitats are often highly variable and interactions between dolphins and their environmental parameters and habitat features are often dictated by the distribution and availability of their prey (Bilgmann et al., 2019). Geospatial analysis of visual sighting data can be helpful to gain insight into hotspots for core biological activities. Additionally, mitigating the impacts of anthropogenic activities requires knowledge about the geographic occurrence of threats (Avila et al., 2018; Cox et al., 2018) and marine mammals' interaction with those threats. Therefore, conservation approaches that use spatially explicit information on marine wildlife populations have the potential to facilitate recovery and contribute to national and international conservation target commitments (Harvey et al., 2017).

The Burrunan dolphins have been previously described as *Tursiops australis* (Charlton‐Robb et al., 2011), an endemic species to south‐eastern Australia, with a distribution from South Australia, eastern Tasmania and Victoria (Bilgmann et al., 2019; Charlton et al., 2006; Charlton‐Robb et al., 2011, 2015; Pratt et al., 2018). The taxonomic status of the Burrunan dolphins, however, is in dispute (see Committee on Taxonomy, 2019; Jedensjö et al., 2017). In Victoria, there are only two known resident populations; one in Port Phillip Bay (PPB) with approximately 150 individuals and the other in Gippsland Lakes (GL) with approximately 60 individuals (Charlton‐Robb et al., 2011). The effective population size (those contributing genes to the next generation) of PPB and GL is 81.5 and 65.5 individuals, respectively (Charlton‐Robb et al., 2015). The Burrunan dolphins are considered vulnerable and at increased risk of decline and/or extinction due to their small population size, genetic distinctiveness, female natal philopatry, exposure to a large degree of associated human and maritime activity and restricted home range, which is in close proximity to a major urban city (Charlton‐Robb et al., 2015; Warren‐Smith & Dunn, 2006).

The Burrunan dolphins were regionally listed as ‘Endangered’ under the Victoria *Flora and Fauna Guarantee Act 1988* in 2013 (Department of Sustainability and Environment, 2013), and have been recently reassessed following the IUCN Red List criteria and the Australian *Environmental Protection and Biodiversity Act 1999* criteria, and is now listed as ‘Critically Endangered’ by the state of Victoria (State of Victoria, 2021). This classification was supported by the population's exposure to numerous anthropogenic threats, such as commercial and recreational fishing, anthropogenic contaminants, tourism, shipping, oil and gas mining, seismic exploration and environmental changes (Charlton‐Robb et al., 2015; Duignan et al., 2020; Filby et al., 2014; Foord et al., 2024; Monk et al., 2014; Puszka et al., 2021). However, the Burrunan dolphins are not classified as threatened (endangered or critically endangered) at a national or global level. It has been documented that the Burrunan dolphins utilise southern PPB (Filby, Christiansen, et al., 2017; Howes et al., 2012; Scarpaci et al., 2003; Warren‐Smith & Dunn, 2006); however, if and how the Burrunan dolphins utilise the whole of PPB (1930 km^2^ in size), and the potential drivers for their distribution, are yet unknown, making the management of the population and mitigation of threats difficult. To this end, this study provides the first assessment of the Burrunan dolphins distribution throughout the whole of PPB, including annual and seasonal variation, and explores possible drivers for the distribution of individuals throughout this environment, providing baseline analysis for conservation recommendations. It further highlights key areas for the consideration of spatial conservation, a critical next step for the effective conservation and management of these regionally threatened populations.

## 2 METHODS

### 2.1 Study site

**FIGURE 1:**
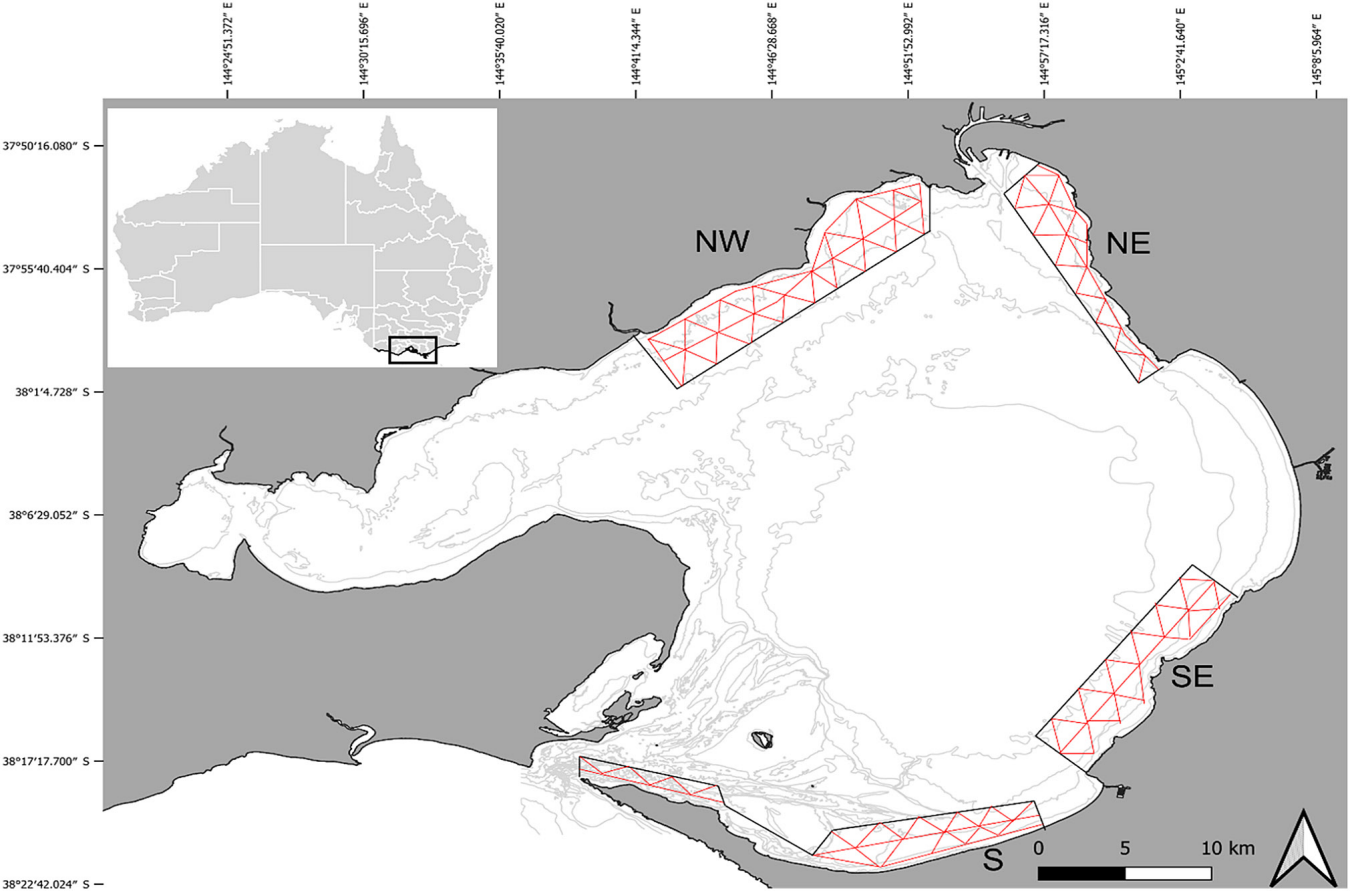
Port Phillip Bay (PPB), with insert showing location within Victoria, Australia. The four survey zones of PPB and the pre‐determined transect line routes (red), NW – north‐west, NE – north‐east, SE – south‐east and S – south.

Port Phillip Bay (Figure 1) is the largest bay (1930 km^2^) in the state of Victoria, Australia, with 333 km of coastline and an average depth of 13 m (Department of Environment, Land, Water and Planning, 2017), which is unusually shallow for its size (Harris et al., 1996). The catchment area of PPB is 9790 km^2^, consisting of 21 natural drainage basins, eight of which deliver runoff directly into the bay. There is limited water exchange due to a narrow, 3‐km wide opening to the Bass Strait (Fu et al., 2017), which results in a flushing time of approximately 12 months (Baker et al., 2016). There are 4.3 million people living within the catchment area of PPB and 1.3 million people living along the coastline (Department of Environment, Land, Water, and Planning, 2017).

### 2.2 Data collection

Seasonal boat‐based surveys were undertaken by the Australian Marine Mammal Conservation Foundation (Marine Mammal Foundation; MMF) from March 2015 to August 2021 across four regions/zones of PPB (Figure 1). Austral seasons were defined as summer (December–February), autumn (March–May), winter (June–August) and spring (September–November). The zones chosen are representative of the entire 1930 km^2^ area of the PPB, covering the southern to the northern reaches (North‐west (NW), North‐east (NE), South‐east (SE) and South (S), and incorporate various habitat types and depths). Some opportunistic sightings did occur outside of these zones. Surveys were conducted during daylight hours, following line transects across the four zones. A 2C research vessel, a 5.7‐m Ensign 570 powered by a 90 hp Mercury engine, was used at survey speeds of 8–12 knots. Surveys were conducted on calm weather days in Beaufort Sea State with conditions of two or less (<15 knot winds), as poorer conditions significantly reduce the detectability of surfacing dolphins.

On each survey, GPS information of the vessel travel path along the transects was recorded using a Garmin eTrex20 handheld GPS, enabling survey effort recording. A crew of three or four researchers conducted constant visual scans across the horizon to sight the Burrunan dolphins. Once dolphins were sighted, the transect was paused, and the research vessel approached the dolphins in accordance with all required scientific research permits and animal ethics guidelines. Photographs of the dolphin's dorsal fins were collected, and behavioural focal points were undertaken during each sighting; however, the use of ID data was outside the scope of this geospatial assessment. Observers on the boat commencing audio recording, dictating the location, environmental conditions and dolphin observation data. Waypoints were recorded at the beginning and end of each dolphin sighting, with vessel movement thus equating to dolphin movement. Sighting observations were deemed complete when observers lost sight of the dolphins and/or the sighting was terminated (e.g., due to poor weather conditions), whereupon the line‐transect was resumed at the point where the vessel left the transect route.

The vessel track of each survey and dolphin waypoints were exported via GPX data and imported into Q GIS 3.10 A Coruna (QGIS Development Team, 2021) to create survey effort maps and to isolate sighting data amongst the survey day tracks. Audio files were transcribed to gain information on each of the sightings for water depth and bathymetry at each 5‐min interval throughout a survey and tallied into eight empirically selected depth contour categories (e.g., 0–5, 5–10 m, etc.) for each sighting.

### 2.3 Data analysis

Dolphin sighting locations were extracted from GPX survey tracks using the position of the vessel at the time of the first sighting of a dolphin (typically within 20 m of the dolphin group). Heatmaps were created using Q GIS 3.10 A Coruna with plugin ‘Heatmap’ (QGIS Development Team, 2021) to display population distribution, zone usage and seasonal and annual variation of the Burrunan dolphins in PPB.

We used generalised linear models (family Poisson) to investigate spatiotemporal variation in the number of sightings during each survey using zone (NW, NE, SE and S) Austral season (summer, autumn, winter and spring) and year (2015–2016, 2018–2021) as factors and effort as a continuous explanatory variable. These statistical analyses were conducted using R Version 4.2.0, in RStudio 2022.02.0 Build 443. To investigate any significant factorial effects on the number of sightings in more detail, including post‐hoc testing, we used package emmeans (Lenth, 2023). For annual comparison, a *p*‐value adjustment using the Tukey method was used to compare a family of six estimates, for season and zone, a *p*‐value adjustment using the Tukey method was used to compare a family of four estimates; the significance level used α = .05.

PPB presents a unique study site to explore whether small incremental bathymetry gradients (minimum 0 m – maximum 40 m) influence marine mammal distribution. Ivlev's selectivity or Jacob's index (Jacob, 1974) was used to evaluate the degree of preference for each depth category:

$$E_{i}=\frac{\left(U_{i}-A_{i}\right)}{\left(U_{i}+A_{i}\right)}$$

where *U*_*i*_ represents the proportion of use of a depth category *i* and *A*_*i*_ its proportional availability. The selectivity index *E*_*i*_ varies from −1 (indicating a use lower than the availability of the category *i*) to 1 (indicating overuse); a value of zero indicates a proportional use of a depth category in relation to its availability.

To visually explore habitat as a potential driver for the distribution of Burrunan dolphins, we used theme layers including Marine and Coastal Feature Atlas, Victorian Biotope Atlas and Planning and Administration from the online data repository CoastKit (Victorian Department of Environment, Land, Water and Planning). These themes provided information about National Parks in PPB and Marine Protected Areas in PPB. The Combined Biotope Classification Scheme (CBiCS) Level 3 Class map (Figure 7) involving 19 different habitat complexes for the study area was developed and provided by the Victorian Department of Environment, Land, Water and Planning for exploration of physical benthic habitat types and communities (Mazor et al., 2021). CBiCS is an ecologically based hierarchical classification system unifying and standardising classifications across marine environments (Edmunds et al., 2021; Edmunds & Flynn, 2015, 2018). The term ‘Biotope’ describes a community of species in a defined abiotic habitat and is used throughout CBiCS; however, as this is not a common term in the literature, hereafter the term used will be ‘benthic habitats’. Desired themes were downloaded as shapefiles and imported into Q GIS. These shapefiles were then overlain with distribution maps of the Burrunan dolphin sighting tracks to display associations of habitat use across PPB.

## 3 RESULTS

**FIGURE 2:**
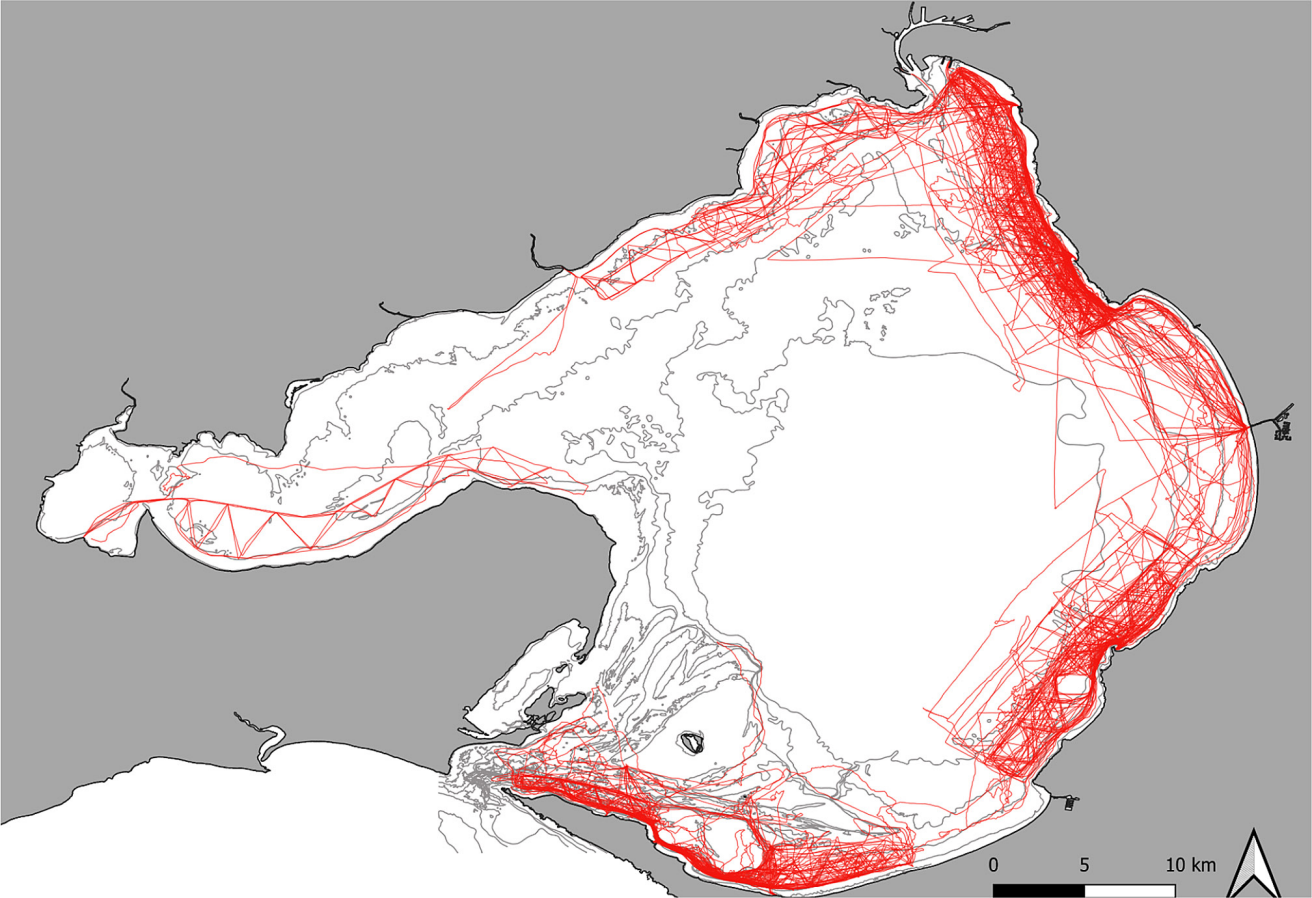
Port Phillip Bay, Victoria, Australia, with survey tracks (red) from March 2015 to August 2021.

During the study period from March 2015 to August 2021, a total of 181 sightings of Burrunan dolphins were recorded across 129 boat‐based survey days in PPB, with 660 hours of survey conducted (Table 1), across all four survey zones, inclusive of vessel transit regions (Figure 2).

**TABLE 1:** The number of Burrunan dolphin sightings, number of survey days and the hours of survey effort across Port Phillip Bay, March 2015 to August 2021.

	Summer		Autumn		Winter		Spring		Total	
	Sightings	Effort (hours)	Sightings	Effort (hours)	Sightings	Effort (hours)	Sightings	Effort (hours)	Sightings	Effort (hours)
2015	0	0 (0)	7	9 (37)	34	17 (87)	0	0 (0)	41	26 (124)
2016	24	10 (44)	13	13 (67)	13	10 (55)	14	7 (41)	64	40 (207)
2018	8	3 (17)	0	4 (23)	5	6 (33)	10	7 (42)	23	20 (114)
2019	3	4 (19)	8	6 (34)	15	7 (41)	7	6 (35)	33	23 (129)
2020	5	4 (21)	2	3 (11)	4	3 (18)	3	4 (14)	14	14 (63)
2021	1	3 (6)	3	2 (11)	2	1 (5)	0	0 (0)	6	6 (23)
Total	41	24 (108)	33	37 (183)	73	44 (238)	34	24 (131)	181	129 (660)

**FIGURE 3:**
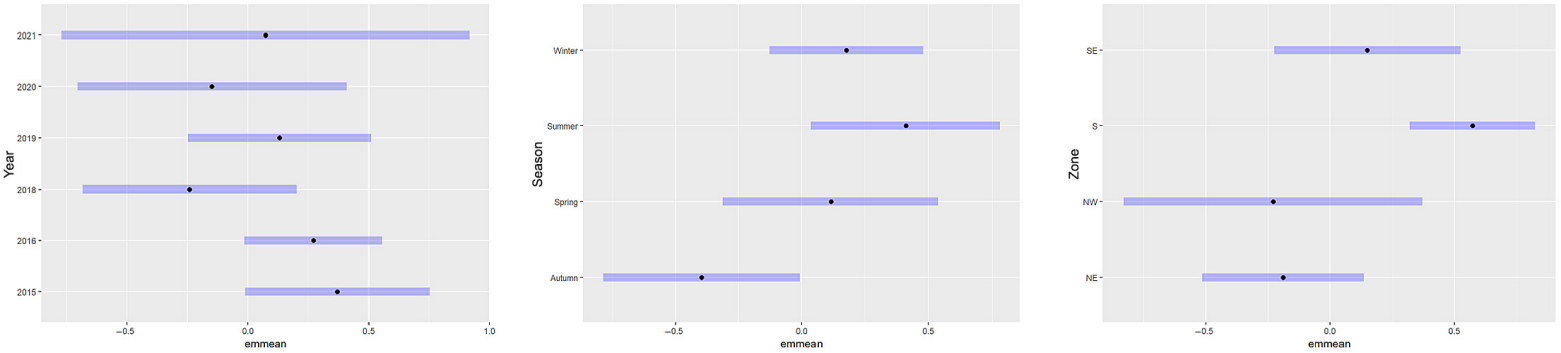
Investigating annual, seasonal and zonal influence of Burrunan dolphin distribution between March 2015 and August 2021 using emmeans. For annual comparison, a *p*‐value adjustment using the Tukey method for comparing a family of six estimates, for season and zone, a *p*‐value adjustment using the Tukey method for comparing a family of four estimates, significance level used α = .05.

Assessment of pooled sightings throughout the 2015–2021 study period found that Burrunan dolphins were observed in all four survey zones in PPB. No annual variation was observed in sighting frequency during 2015–2021, with no differences observed between individual years. Emmeans graphs showed some seasonal variation in the sighting frequency of Burrunan dolphins. Autumn had a lower number of sightings than both summer and winter (*p*‐value = .01 and .04, respectively), with spring being indistinguishable from other seasons (Figure 3). Zonal variation was also observed, with the S zone having a higher number of sightings than the NW and NE zones (*p*‐value = .05 and <.01, respectively) (Figure 3).

**FIGURE 4:**
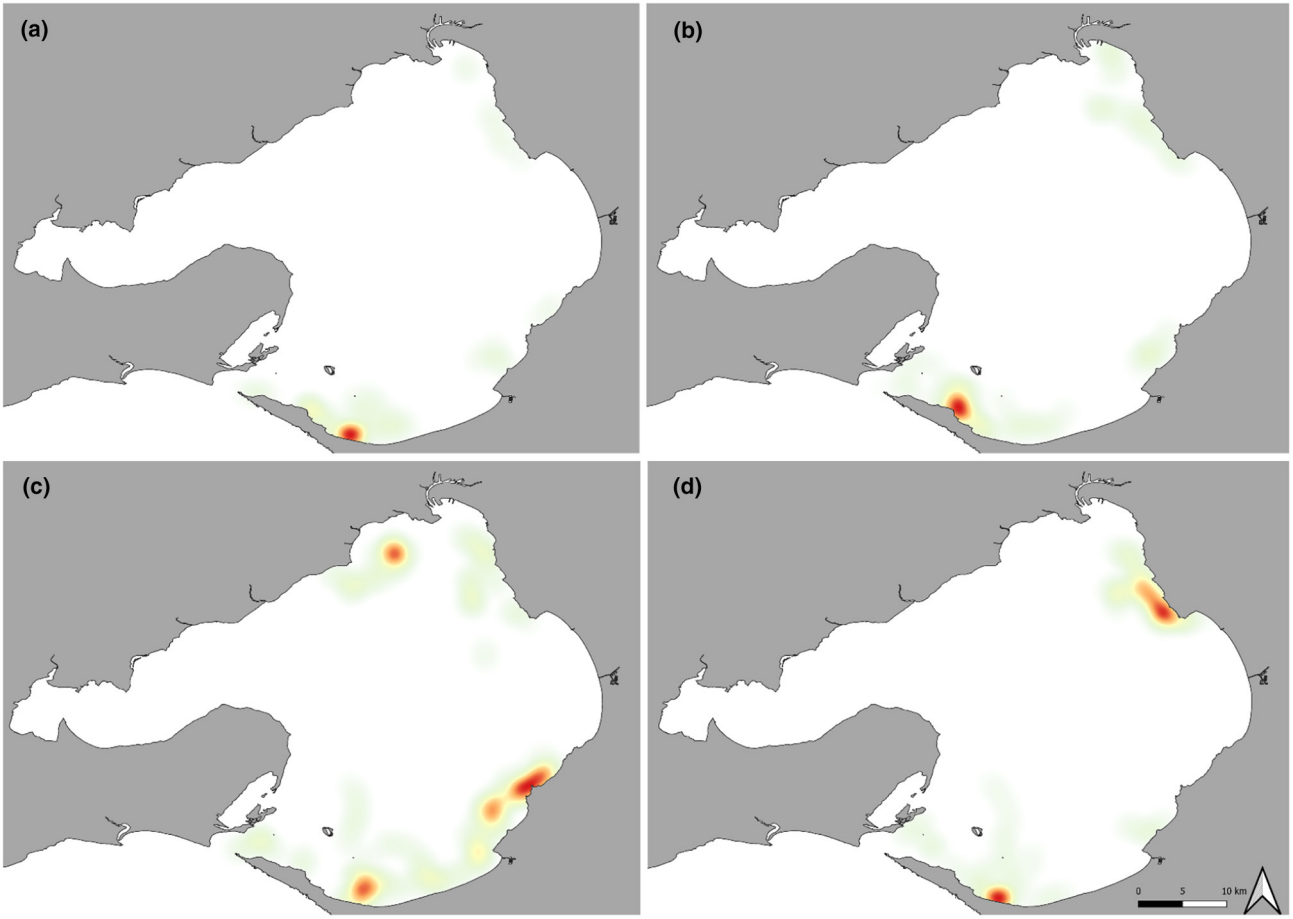
Seasonal heatmaps of Burrunan dolphin sightings from March 2015 to August 2021 in Port Phillip Bay, Victoria, with colours graduating from areas of high sightings (red) to areas of low sightings (green), (a) Summer, (b) Autumn, (c) Winter and (d) Spring.

Seasonal movement patterns were observed throughout the 2015–2021 survey period. Dolphin sightings were higher within the S zone of PPB in summer (December–February) and autumn (March–May), whilst a wider region of PPB was utilised during winter (June–August) and spring (September–November) (Figure 4).

**FIGURE 5:**
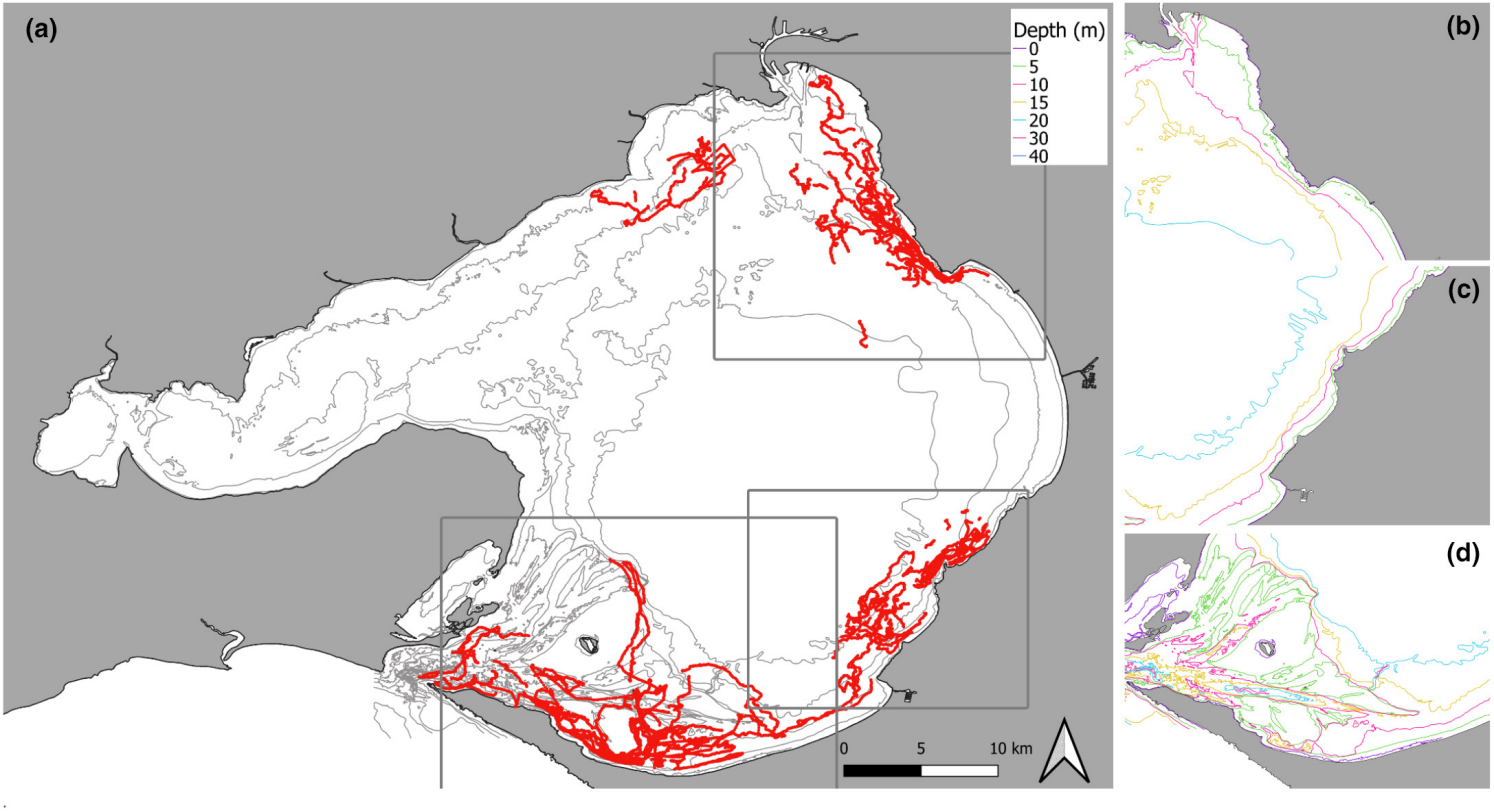
(a) Burrunan dolphin sighting tracks (red) from March 2015 to August 2021 with bathymetry contours of Port Phillip Bay (grey). Inserts showing greater details in high sightings areas with colour coded bathymetry depths, (b) NE zone, (c) SE zone and (d) S zone.

Dolphin sighting tracks closely follow bathymetric contour lines (Figure 5) in both the north and south of PPB. The NE zone, SE zone and the southern region of PPB displayed high dolphin sightings; these regions also had complex bathymetrical contours (Figure 5).

**FIGURE 6:**
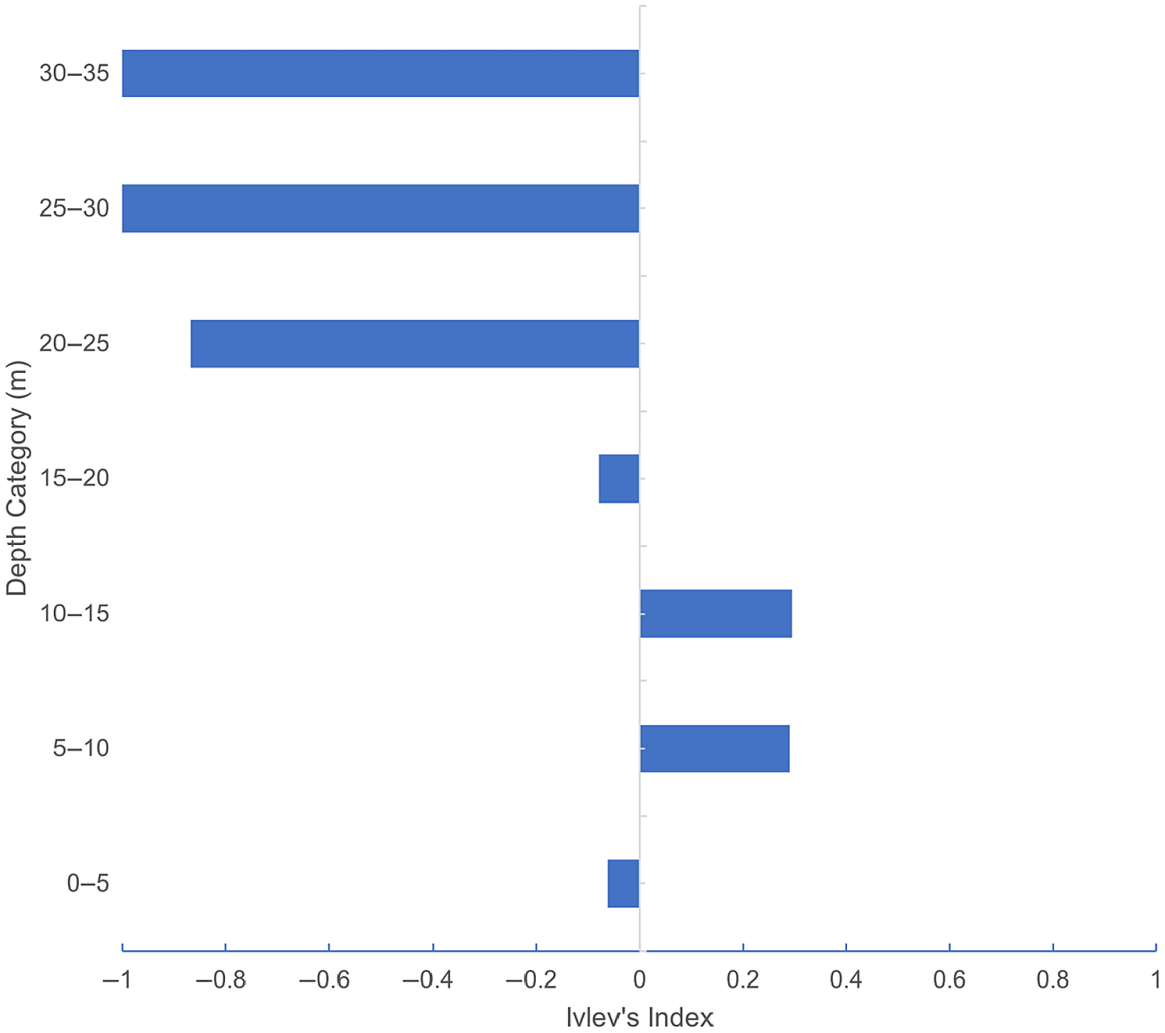
Burrunan dolphin depth contour preference between 0 and 35 m from March 2015 to August 2021 (Ivlev's selectivity index).

The preference for Burrunan dolphins to use particular depth contours was explored using Ivlev's selectivity index (Figure 6). The Burrunan dolphins showed preference for the 5–10 and 10–15 m depth categories (*I* = 0.29 and 0.30, respectively). Furthermore, the Burrunan dolphins avoided areas of depth lower than 5 m and greater than 20 m (*I* = −0.06 and −0.87, respectively).

**FIGURE 7:**
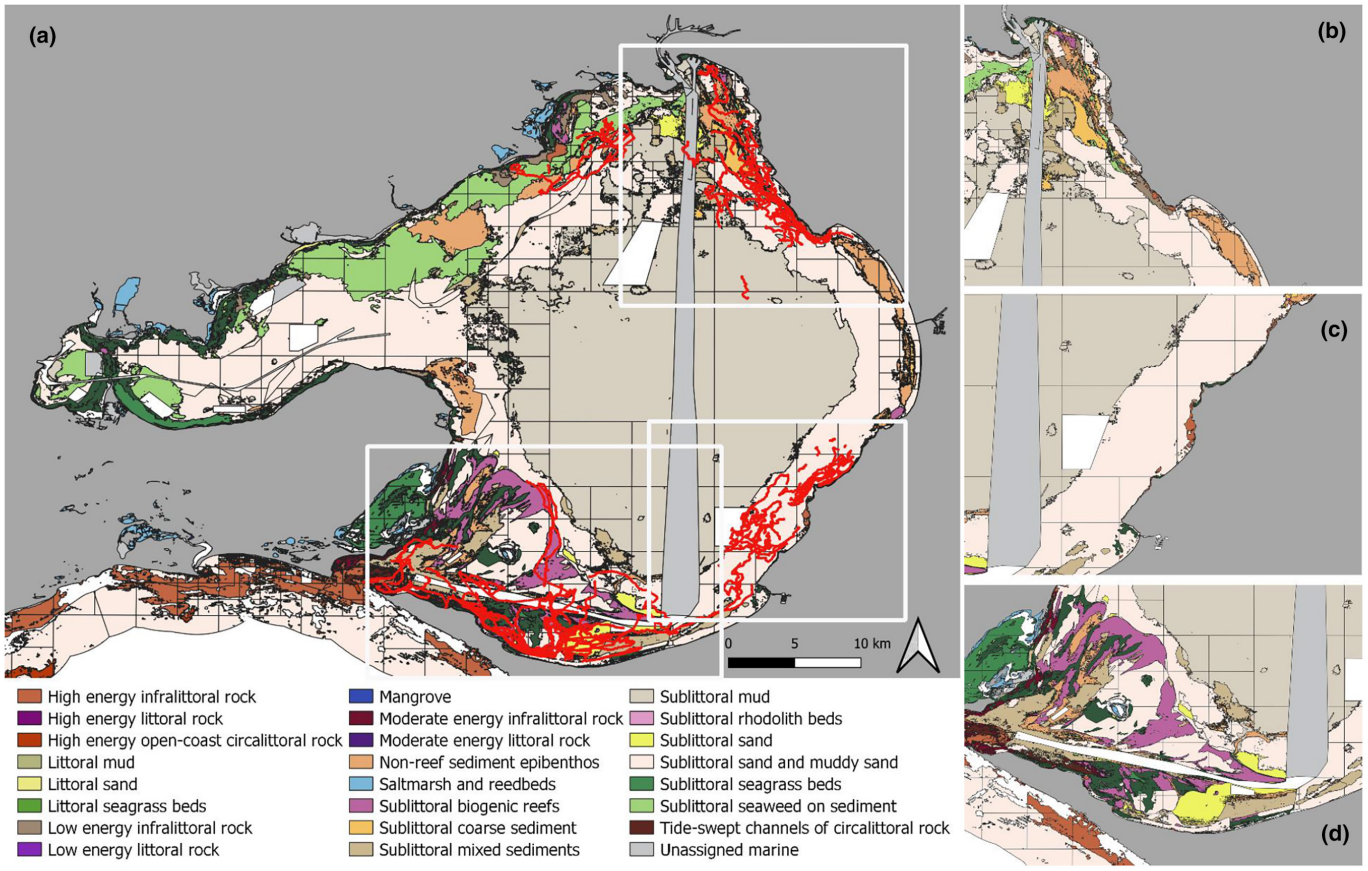
(a) Burrunan dolphin sighting tracks (red) from March 2015 to August 2021 overlain with Port Phillip Bay biotope regions. Inserts showing greater details in high sightings areas, (b) NE zone, (c) SE zone and (d) S zone.

Using the CBiCS classification and seagrass layers to create exploratory maps, areas of high dolphin sighting tracks were seen around several benthic habitats, in particular sublittoral seagrass beds, sublittoral rhodolith beds and high and low energy infralittoral rock regions that transition into sublittoral mud and sand regions (Figure 7). These regions correspond with areas of bathymetry complexity (Figure 6). There was a high number of sightings around the transitional boundaries of benthic habitats.

**FIGURE 8:**
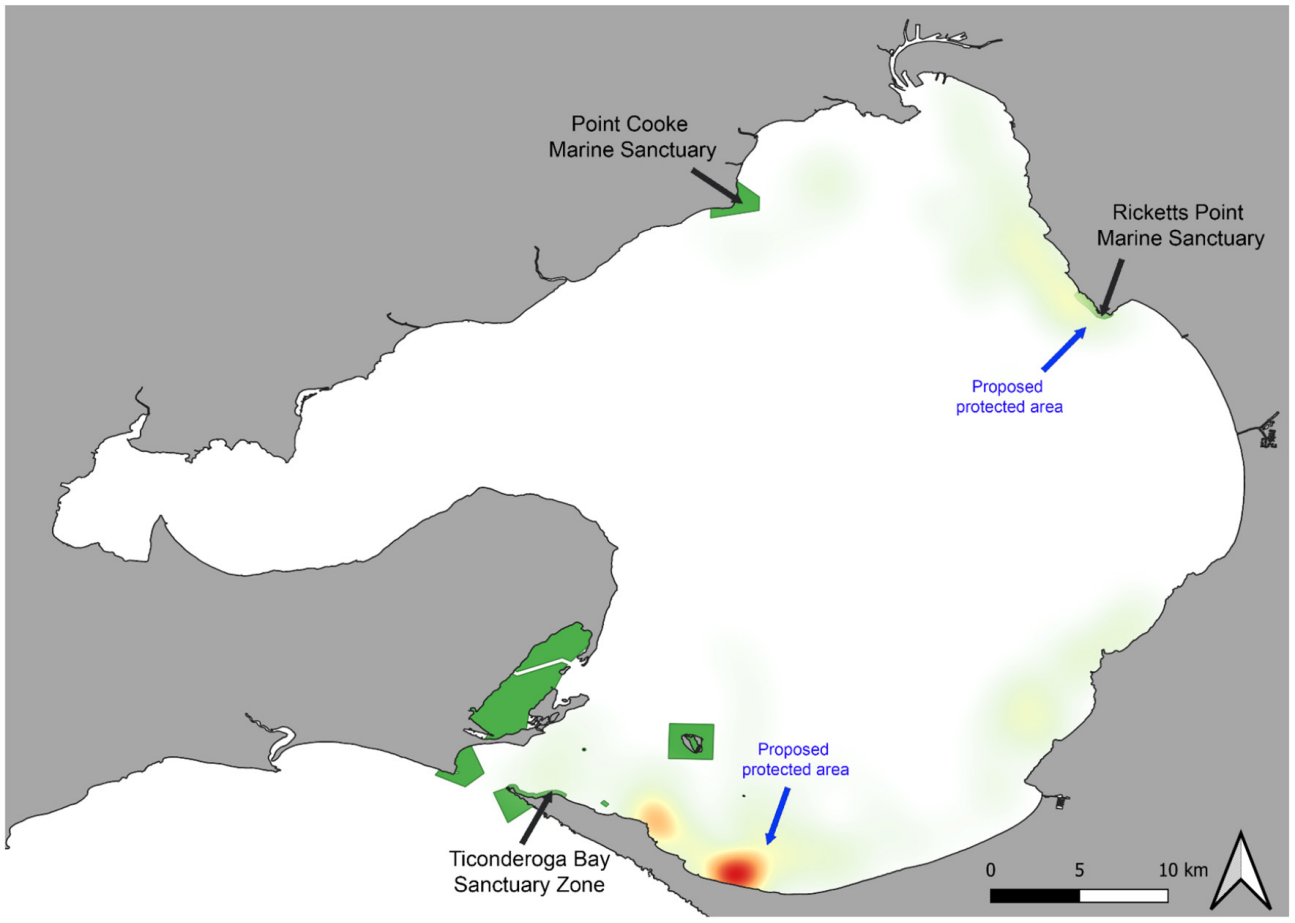
Burrunan dolphin pooled sighting from March 2015 to August 2021, represented as heatmaps with colours graduating from areas of high sightings ‘hotspots’ (red) to areas of low sightings (green), overlain with the Marine National Parks within Port Phillip Bay (green) and proposed protected areas (blue arrow).

Of the four marine parks and sanctuaries across PPB, the Burrunan dolphins frequented the Ricketts Point Marine Sanctuary in the NE zone (Figure 8). None of the sightings seen in the NW zone overlapped with the Point Cooke Marine Sanctuary. Few sightings were seen within the boundary of the protected areas within the Port Phillip Bay Heads Marine National Park, and three sightings were seen within the Ticonderoga Bay Sanctuary Zone.

## 4 DISCUSSION

Baseline information on the distribution and movement patterns of a population is critical for effective conservation and management of wildlife. The analysis of population distribution patterns at a fine‐scale provide the best resolution for examining local species‐environment relationships, habitat usage and anthropogenic impacts (Brough et al., 2019; Harwood et al., 2014; Zanardo et al., 2016). Food availability, predation risk and anthropogenic activities are known to influence delphinid habitat use (Heithaus & Dill, 2006; Pirotta et al., 2019). Using sightings data collected during 2015–2021 from four survey zones representative of numerous benthic habitats and bathymetrically complex areas in PPB, we found that the Burrunan dolphins utilise the entirety of PPB, from the northern to the southern reaches, show seasonal distribution changes and have higher sightings in regions of complex bathymetry. Sublittoral seagrass beds, sublittoral rhodolith beds and high‐ and low‐energy infralittoral rock regions that transition into sublittoral mud and sand regions were found to have a high number of sightings also.

### 4.1 Seasonal variation in Burrunan dolphins distribution

Marine animal populations tend to shift their geographic ranges in response to varying environmental conditions, resulting in seasonal shifts in population distribution. In particular, dolphin associations with environmental parameters and habitat features are often dictated by the distribution and availability of prey (Bilgmann et al., 2019; Hastie et al., 2004; Heithaus & Dill, 2002; Rayment et al., 2010). Seasonal shifts in population distribution provide an additional challenge for the spatial protection of dolphin populations. We found consistent sightings across all seasons in the southern region, and a northwards trend in the Burrunan dolphins distribution was observed during the winter (SE zone, Figure 4) and spring (NE zone, Figure 4). We hypothesise that a subgroup of the population displays high site fidelity within the southern zone, remaining in the area year‐round, whilst another subgroup of the population migrates north in winter and spring. Higher density of sightings across more zones during winter and spring indicates a broader region of PPB is being utilised throughout these seasons. Individual or small group movement patterns within a population can also vary greatly, as some members of a resident population may remain within a small home range (Gubbins, 2002; Zolman, 2002), whereas other members of the same population may display little preference for a particular area (Toth et al., 2011). Shane et al. (1986) suggested that ranging patterns of populations can vary from permanent local ranges to seasonal migration to short‐term seasonal site fidelity. Confirmation of this high site fidelity perceived in certain subgroups is critical for effective conservation management, ensuring that the subgroup has a protected area to continue core biological activities and respite from anthropogenic activities such as high vessel traffic. Ongoing research is required to confirm this hypothesis, with continued boat‐based surveys and photographic dorsal fin identification of individuals within the PPB population. This robust identification methodology would allow for individual and potential subgroup site fidelity and movement patterns to be further investigated.

Throughout all seasons, the southern region of PPB experienced a high dolphin presence (Figure 3). A decline in dolphin presence was previously hypothesised during the summer period due to increased recreational boat traffic and tour operator activity (Filby et al., 2014; Scarpaci et al., 2003); however, this was not evidenced during the current study. Four swim‐with‐dolphin tour vessels operate within the region, with each vessel running a maximum of two trips daily between October and May (Filby, Christiansen, et al., 2017). Filby et al. (2014) highlight the impacts of tour vessels and recreational boat interactions on dolphin behaviour, with these interactions often resulting in the expenditure of greater amounts of energy avoiding vessels in the dolphins impacted. Dolphins may remain in an area of high vessel disturbance while altering behaviour to minimise the disturbance (Lusseau, 2003; Williams et al., 2004). For example, they may temporarily move away during periods of high vessel activity but return once vessel traffic has reduced, or they may abandon a region that was once preferred due to vessel disturbances (Bejder et al., 2006). These impacts can indirectly affect the fecundity and survival of the population (Gill et al., 2001; Steckenreuter et al., 2011). Despite the biological cost linked to these human impacts, the Burrunan dolphins remained in the southern region during peak vessel activity (Figure 3). The Indo‐Pacific bottlenose dolphin population found in Port Stephens, NSW (Steckenreuter et al., 2012; Wiszniewski et al., 2009, 2010) exhibited changes in their activity budgets in the presence of boats, with no resting, reduced feeding and socialising recorded and an increase in milling and travelling behaviours when boats were in the area. Similarly, the Burrunan dolphins spent less time foraging when swim‐with‐dolphin tourism vessels were present (Filby, Christiansen, et al., 2017). Unfortunately, the PPB population may not be able to avoid anthropogenic disturbance by moving away from the current habitat, as they have likely adapted to the environmental and ecological conditions of the area.

A potential driver for the observed seasonal shifts in the contraction and expansion of distribution ranges could be in response to breeding and/or birthing seasons (Clutton‐Brock, 1997; Greenwood, 1980; Sprogis et al., 2016). Long‐term studies of bottlenose dolphin populations have found that females tend to have a smaller home range and stronger site fidelity (Gubbins, 2002; Smith et al., 2013; Urian et al., 2015; Wells, 2003), while males tend to have lower site fidelity and larger home ranges, especially during nonbreeding seasons when they may adjust their home ranges to optimise prey intake (Sprogis et al., 2016, 2018). The age and sex of individuals included in this study are unknown; however, further investigation into the sex‐specific distribution of the Burrunan dolphins would be beneficial for supporting the sub‐group hypothesis previously mentioned.

Another potential driver for the observed seasonal shifts of distribution, and potential subgroup movement patterns, is prey availability. Information regarding the diet of the Burrunan dolphin is limited, although through observation and isotope analysis, the dolphins are thought to feed on garfish, calamari squid, snapper, sand flathead, yellowfin bream and barracouta (Filby, Stockin, & Scarpaci, 2017; Owen et al., 2011) (Appendix S1). Further to this, the examined contents of deceased PPB dolphins suggest that King George whiting and Australian salmon may also occur within their diet (Mason, 2007 as cited in Filby, Stockin, & Scarpaci, 2017). In comparing the high prevalence of sightings across all seasons in southern PPB with potential prey resources, we note peak spawning of southern calamari occurs in nearshore habitats typically between spring and early summer; however, this can occur all year round (Moltschaniwskyj & Steer, 2004; Smith et al., 2015). Spawning typically occurs in inshore coastal regions, with eggs laid in seagrass and algal reef habitats (Commissioner for Environmental Sustainability, 2021). Garfish have also been observed in deep seagrass regions in February and March within the south of PPB (Smith et al., 2012). We suggest that increased foraging occurs at the southern end of PPB due to bathymetry complexity, seagrass regions and prey availability, especially during the summer. However, it is also possible, given the close proximity of this region to Bass Strait, Burrunan dolphins may travel outside of PPB in search of resources. Stable isotope analysis conducted by Owen et al. (2011) found that the PPB population was 4.5% higher in δ^15^N than the average signature of potential prey items within PPB. This suggests that the PPB population have additional unidentified prey resources that have a higher trophic level than that of the prey items sampled. This supports the hypothesis that a subgroup of Burrunan dolphins shows high site fidelity to the southern region of PPB all year round, and when resources inside the southern end of PPB are no longer sufficient, they may forage in Bass Strait and then utilise the inside of the bay in the southern region for other core biological activities (e.g., milling/resting and social activities).

Port Phillip Bay also experiences a major immigration of larger reproductive snapper during spring and summer, which have a limited summer spawning period (Hamer & Jenkins, 2004). High sightings of Burrunan dolphins in the NE zone (Figure 4) correspond with the peak recreational fish catch seen in late winter and spring, and this is driven by snapper movement into northern regions PPB in anticipation of spawning season (Hamer et al., 2011; Longmore, 2014; Ryan et al., 2019). The shallow reefs along the NE coastline have been identified as highly suitable for subadult snapper (Morris & Ball, 2006). In addition, spawning for King George whiting occurs near coastal reefs in autumn and early winter (Fowler et al., 2000; Jenkins & King, 2006), which spend their first 4 years maturing in the PPB seagrass nurseries (Commissioner for Environmental Sustainability, 2021). It is therefore likely that the numerous potential prey species aggregating in higher abundance in the northern reaches of the bay during this period, may enable greater foraging opportunities for the Burrunan dolphins. The seasonal distribution of these prey items supports the hypothesis of a subgroup migrating north to the SE zone during winter and moving further north to the NE zone during spring to follow prey resources. Further research investigating the foraging behaviour and prey choice of the Burrunan dolphins, both inside of PPB and possibly in the Bass Strait region, is encouraged to help identify other key regions of Burrunan dolphin distribution and confirm the population's specific diet preferences.

### 4.2 Oceanographic drivers of distribution across Port Phillip Bay

Bathymetry is an important variable in explaining habitat distributions, as it acts as an indirect proxy of light availability (Ierodiaconou et al., 2018), which in turn may directly or indirectly affect zooplankton, fish phenology (Durant et al., 2019) and benthic communities (Douglas et al., 2022; Rovelli et al., 2019). Bathymetric variability and bottom structure can lead to increased biological productivity (Simard et al., 2015); surface complexity can also influence the availability of food, protection from predation, exposure to currents and wave action (Ierodiaconou et al., 2007). Studies have found that water depth and bathymetrical complexity are significant factors in determining the distribution of marine species (Gross et al., 2009; Hastie et al., 2004, 2005), with bathymetry strongly associated with patterns of marine mammal species richness and complementarity (Astudillo‐Scalia & de Albuquerque, 2020). A high association between the Burrunan dolphins' movement patterns and the bathymetric contour lines was found (Figure 5), with sighting tracks often running parallel with bathymetry lines. These high sighting areas correlate to gradients in depth, with gradients from 5 to >15 m occurring close to shore (Figure 5), creating a complex bathymetry environment. Complex landscapes can facilitate prey capture by providing physical barriers to corral prey, slowing down the escape of prey and providing predator stalking cover (Bouchet et al., 2015; Chundawat, 1990; Sweanor et al., 2000). Orcas in the Pacific Northwest have been found to herd prey using bathymetric features, by driving fish towards physical barriers to concentrate the prey into denser groups (Bouchet et al., 2015; Heimlich‐Boran, 1988). This is similar to the PPB Burrunan dolphin, which has shown a preference for complex bathymetrical regions, likely to aid with prey capture.

The PPB Burrunan dolphin population provides an insight into the effects of depth as a driver of distribution in a shallow coastal environment. We show evidence of the selection‐avoidance of habitat types of differing depths, with the 20–25 m depth category being utilised in a significantly lower proportion relative to the percentage of this depth category within PPB (27%). Burrunan dolphins were found to prefer the 5–10 and 10–15 m depth contours. Our findings are similar to shallower water depth preference and/or occurrence of Australian humpback dolphin, Ningaloo Marine Park (Western Australia) 5–10 m (Hunt et al., 2020); the southern Australian bottlenose dolphin, Coffin Bay (South Australia), 2–4 and 7–10 m (Passadore et al., 2018); and the Indo‐Pacific humpback dolphin Bay of Bengal (India) 5–15 m (Lin et al., 2021). In each of these studies, prey and predator avoidance have been the most commonly documented drivers for these depth preferences. Areas at these particular depths or gradients have been documented to improve accessibility to demersal fish, and the regional bathymetry profile could positively affect the handling efficacy of catching prey (Durden et al., 2019; Hastie et al., 2003, 2004; Wang et al., 2021; Wu et al., 2017). In this case, demersal fish, such as snapper and King George whiting, are documented prey items of Burrunan dolphins (Filby, Stockin, & Scarpaci, 2017; Owen et al., 2011). There is little peer‐reviewed documentation on the preferred depths of the Burrunan dolphin's prey species in PPB. Trawls conducted by Parry et al. (1995) found the largest biomass of snapper at depths of 12 m, and anecdotal evidence describes King George whiting's preferred depth as 3–10 m. In an adjacent Victorian embayment, Western Port Bay, the highest proportion of snapper was found between 7 and 18 m, and the King George whiting was found within 2–10 m (Jenkins et al., 2020). Therefore, we loosely hypothesise, based on the limited data, that the Burrunan dolphin's preference for 5–10 and 10–15 m depth contours may be associated with prey availability and capture; however, more research is required to explore this theory.

Benthic habitat, or particular habitat regions (e.g., soft sediment, seagrass rock and reefs) play an important role as predictors of a species' spatial distribution. A wide range of benthic habitats have been thought to drive dolphin distribution around the world (Bennington et al., 2021; Bonneville et al., 2021; Gross et al., 2009; Sprogis et al., 2022; Zanardo et al., 2017). South Australian bottlenose dolphins were found to have a year‐round preference for bare sand habitat; however, preference for seagrass regions was seen to increase during summer and autumn, which could be indicative of a seasonal variation in habitat preference (Cribb et al., 2013; Zanardo et al., 2017). Alternatively, Indo‐Pacific bottlenose dolphins in the coastal areas of Noumea and Plum were found to favour muddy bottoms (Bonneville et al., 2021). Whereas, Indo‐Pacific bottlenose dolphins in Bunbury southwestern Australia were found to have a preference for reef habitat, followed by a preference for sand and mud/silt (Sprogis et al., 2018). These regions likely constitute the habitat regions where the prey of the coastal dolphin are concentrated (Gross et al., 2009).

This study showed a higher number of sightings of Burrunan dolphins in sublittoral seagrass beds, sublittoral rhodolith beds and high‐ and low‐energy infralittoral rock regions that transition into sublittoral mud and sand regions, indicating these regions provide suitable habitat for the dolphins and/or potential prey items. Seagrass beds and rhodolith beds have greater fish diversity and density than adjacent flattened areas (Costa et al., 2020; Heck et al., 1997; Horta et al., 2016). These zones also provide nursery grounds for many fish assemblages (Madi Moussa et al., 2020; Verweij et al., 2008), including some of the Burrunan dolphin prey species such as snapper (Owen et al., 2011), King George whiting and squid (Filby, Stockin, & Scarpaci, 2017). It is often assumed that dolphins feed primarily within seagrass beds, as these habitats are where fish are most abundant (Wilson et al., 2017). However, recent studies have suggested that these environments may hinder foraging, as seagrass attenuates echolocation and fish vocalisations by scattering sound energy (Wilson et al., 2013). Therefore, dolphins may prefer to forage in less dense seagrass patches (Mann et al., 2021) or on the edge of seagrass beds, in transitional zones (Allen et al., 2001; Nowacek, 2005), where acoustic detection of prey is more efficient. Infralittoral reefs are key habitats for many fish species because they can provide a source of food and shelter (Davis et al., 2020; Young et al., 2022). Anderson (2003) also found that sand‐associated fish species such as sand flatheads were more common in close proximity to structured rather than completely unvegetated habitats, which further supports findings from Ferrell and Bell (1991) that non‐seagrass fish species are more abundant in sand within 10 m of seagrass (Smith et al., 2008). Juvenile snapper has also been found to be most abundant over soft sediments that are adjacent to rocky reef areas, preferring reef‐sand boundaries (Langlois et al., 2005; Rees et al., 2021; Ross et al., 2007). As these are both prey species, this may explain why Burrunan dolphins are frequently sighted in these transitional zones (infralittoral rock to sublittoral mud and sand, and seagrass to sand regions). Overall, benthic habitats appear to play a key role in driving the distribution of Burrunan dolphins in PPB, in combination with other factors.

### 4.3 Marine protected areas

Port Phillip Bay has four Marine National Parks and Sanctuaries; however, only one dolphin sanctuary zone is specified for the ‘protection’ of dolphins, located in southern PPB (Figure 8). Ticonderoga Bay Sanctuary Zone (TBSZ) was established in 1996, aiming to provide respite and refuge for resident Burrunan dolphins (Howes et al., 2012) through the introduction of stringent approach and speed regulations in place for vessels (Department of Environment, Land, Water, and Planning, 2019). However, as stated by Filby, Stockin, and Scarpaci (2017), the implementation of this sanctuary zone was not based on robust scientific observational data; rather, the proposal was based on anecdotal dolphin observations in the area, which did not reveal whether TBSZ was of critical importance to the population in terms of usefulness for core biological activities (Filby, Stockin, & Scarpaci, 2017) and was lacking scientific validation (Howes et al., 2012). As TBSZ is the only designated protected area for the Burrunan dolphins in PPB, we explored how the sightings observed in this study compared with the overall region. Of the 181 sightings that occurred during 2015–2021, only three sightings were observed in the TBSZ. This raises further questions about whether this one sanctuary zone is effective for the conservation management of the species. A much higher density of dolphin sightings was noted in the southern zone, further east of TBSZ (Figures 4 and 8). This higher sighting density area is of particular concern as anthropogenic activities intensify during the summer period in this location, with an increased number of recreational vessels, tour boat operations and swim‐with‐dolphin tourism. This peak in human activities is likely to overlap with Burrunan dolphin habitat use and potentially cause disturbances to the population.

Further, the Ricketts Point Marine Sanctuary, established in 2002 in the north‐eastern PPB, appears to be a habitat ‘hotspot’ zone for the Burrunan dolphin population, showing a high sighting density (Figures 4d and 8). Ricketts Point Marine Sanctuary covers 115 ha, within which fishing is prohibited. In the shallows of the marine park, there are seagrass beds that form nurseries and feeding grounds for many animals. Australian marine reserves were found to have a 28% greater abundance and 53% greater biomass of fished species compared to open fishing areas (Goetze et al., 2021). Additionally, many MPAs can produce ‘habitat spillover’ where species from inside the protected area move to surrounding unprotected areas (Forcada et al., 2009), and can be seen to benefit areas adjacent to implemented MPAs. The benefits of marine reserves were greater in highly protected (no‐take reserves), like Ricketts Point Marine Sanctuary, and increased with size, age, connectivity and depth (Goetze et al., 2021). In this study, we see evidence of this, with a high number of dolphin sightings in and around the Ricketts Point Marine Sanctuary zone. As such, it is likely to be a worthy candidate for further protection. We recommend expanding the sanctuary borders to the 15 ‐m depth contour line to increase the overall size and depth range incorporated in the sanctuary zone.

Overall, we found that the Burrunan dolphins used areas within and around existing protected areas. However, other core areas of high use still remain unprotected. As the Burrunan dolphins are regionally listed as a critically endangered species by the state of Victoria (State of Victoria, 2021), the successful implementation of MPAs (and marine mammal‐specific MPAs) is critical for this population's survival. The Burrunan dolphins distribution and habitat use were found to have a low association with the TBSZ, with only three sightings within the zone throughout the 6 years of survey. As such, we recommend that TBSZ remain a dedicated dolphin sanctuary zone until further research into the area is conducted. Two additional areas of PPB were identified as critical ‘hotspot’ zones and areas of importance for the Burrunan dolphins; the southern zone of PPB to the east of TBSZ and the NE zone of PPB, near the Ricketts Point Marine Sanctuary. Given the known anthropogenic threats impacting the species, and of particular note, Filby, Stockin, and Scarpaci (2017) and Puszka et al. (2021) observing behavioural impacts in the dolphins in response to vessel interactions, we recommend affording these two additional areas the same level of protection as TBSZ (no approach of vessels within 200 m, no approach of jet skis within 300 m, 5 knots speed limit in the zone), allowing for greater protection of the Burrunan dolphins in both habitat ‘hotspots’. This would see the creation of a new MPA in the southern zone, which we recommend to provide year‐round protection for the Burrunan population since they are seen in the southern zone all throughout the year (Figures 4 and 8). In the NE zone, we recommend the expansion of Ricketts Point Marine Sanctuary. This expansion could be a seasonal protection that considers the influx of Burrunan dolphins into the NE zone during spring (Figure 4).

## 5 CONCLUSION

For the first time, this study investigates the distribution and habitat use of the Burrunan dolphins throughout PPB, greatly increasing our understanding of species presence across PPB. The distribution of Burrunan dolphins was seen to vary seasonally, with prey resources presumably acting as a seasonal driver. The observed seasonality of sightings also inferred potential subpopulation site fidelity, with dolphin presence year‐round in southern PPB, and more wide‐spread distribution during winter and spring. Further, we found the dolphins favouring certain depth contours (5–10 and 10–15 m) and benthic habitat transitional zones (sublittoral seagrass beds, sublittoral rhodolith beds and high‐ and low‐energy infralittoral rock regions that transition into sublittoral mud and sand regions). As the impacts of human activities may threaten the survival of this species, we recommend two additional dolphin sanctuary zones be established to serve as critical habitat hotspots for the population. This includes the addition of a new static sanctuary zone in the southern zone of PPB and the seasonal expansion of the current Ricketts Point Marine Sanctuary. We provide a baseline PPB‐wide distribution study and recommend continued monitoring in the current zones and exploration into other undocumented areas across PPB based on these findings, as well as for the implementation of successful conservation management strategies for the protection of the Burrunan dolphins.

## AUTHOR CONTRIBUTIONS

**Jemima Beddoe:** Conceptualization (equal); data curation (equal); methodology (equal); project administration (equal); visualization (equal); writing – original draft (lead); writing – review and editing (equal). **Jeff Shimeta:** Conceptualization (equal); methodology (equal); project administration (equal); supervision (equal); writing – review and editing (equal). **Marcel Klaassen:** Formal analysis (equal); methodology (supporting); writing – review and editing (equal). **Kate Robb:** Conceptualization (equal); funding acquisition (lead); methodology (equal); project administration (equal); resources (lead); supervision (equal); writing – review and editing (equal).

## CONFLICT OF INTEREST STATEMENT

We have no conflict of interest to declare.

## Supporting information


Appendix S1.

Data S1:

## Data Availability

The data that supports the findings of this study are available in the supplementary material of this article.
