# Supplementary material for: Population distribution and drivers of habitat use for the Burrunan dolphins, Port Phillip Bay, Australia
Source: Ecol Evol. 2024 Apr 4;14(4):e11221. doi: 10.1002/ece3.11221 (PMC10994984; doi:10.1002/ece3.11221)
Supplement: Supplementary file 1 — Appendix S1. [file ECE3-14-e11221-s001.docx]

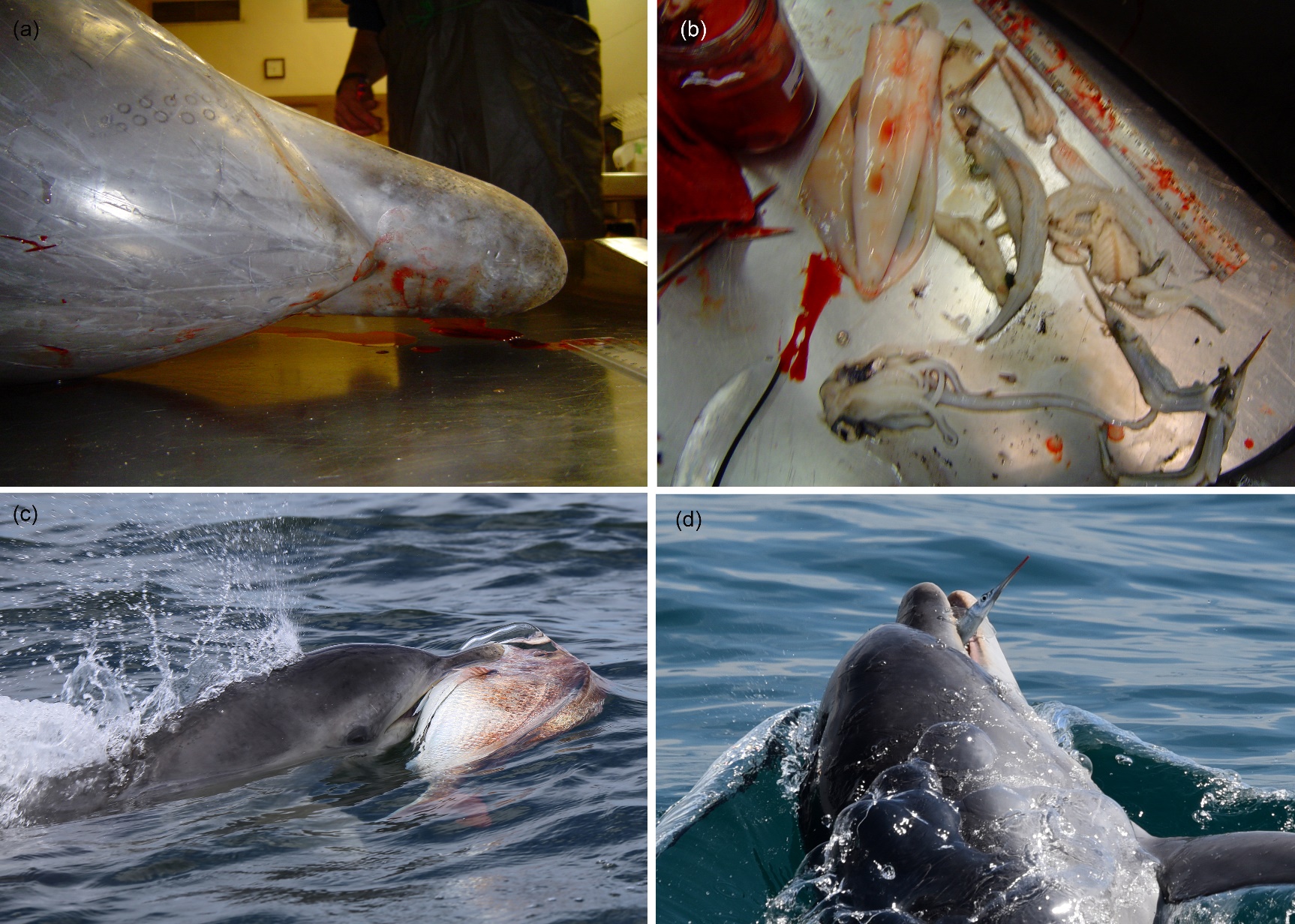


Appendix S1. Port Phillip Bay Burrunan dolphin with prey items a) calamari squid sucker marks, b) stomach content of deceased Burrunan dolphin, calamari squid and garfish, c) snapper, b) garfish. Photo credited to the Marine Mammal Foundation.
